# How does the delivery of paid home care compare to the care plan for clients living with dementia?

**DOI:** 10.1111/hsc.13761

**Published:** 2022-02-22

**Authors:** Pat Brown, Monica Leverton, Alexandra Burton, Karen Harrison‐Dening, Jules Beresford‐Dent, Claudia Cooper

**Affiliations:** ^1^ Dementia UK London UK; ^2^ NIHR Health & Social Care Workforce Research Unit Kings College London London UK; ^3^ Department of Behavioural Science and Health University College London London UK; ^4^ Division of Psychiatry University College London London UK

**Keywords:** care planning, dementia, ethnography, home care, independence, person‐centred care, qualitative research

## Abstract

Many people living with dementia choose to remain in their own homes, supported by home‐care workers, who provide care that is specified in care plans. We explored how care plans of clients living with dementia, compared with ethnographic observations of home care they received. In a secondary, reflexive thematic analysis, we reviewed care plans for 17 clients living with dementia and transcripts from 100 h of observations with 16 home‐care workers delivering care to them. Our overarching theme was: *Care plans as a starting point but incomplete repository*. Clients’ care plans provided useful background information but did not reflect a wealth of knowledge home‐care workers built through practice. Two sub‐themes described: (a) *Person‐centred care planning*: whether and how the care plan supported tailoring of care to clients’ needs and (b) *Filling in the gaps*: home‐care workers often worked beyond the scope of vague, incomplete or out‐of‐date care plans. We found considerable inconsistencies between care plans and the care that was delivered. Care plans that were comprehensive about care needs, and rich in person‐specific information aided the delivery of person‐centred care. Lack of documentation was sometimes associated with observed failures in person‐centred care, as helpful information and strategies were not shared. Including information in care plans about how, as well as what care tasks, should be completed, and frequently discussing and updating care plans can create more person‐centred plans that reflect changing needs. Electronic care planning systems may support this.

**What is known about this topic?**

- Home‐care services support over 350,000 older people in the UK.
- Sixty percent of UK home‐care workers work with people living with dementia.
- Domiciliary care organisations rely on care plans to inform the care they provide to people living with dementia.

**What this paper adds?**

- The care observed often differed in important ways from the instructions within the care plan of the person living with dementia because the care plans had not been updated for some time.
- Whilst some care plans gave explicit instructions to guide person‐centred care, in many, guidance was generic and untailored to the client. This left home‐care workers to ‘fill in the gaps’—often creatively, but this learning was not documented and therefore unavailable to the wider home‐care team.
- Our findings suggest that there may be a role for more dynamic information‐sharing systems in supporting the implementation of person‐centred care within the home‐care sector; newer electronic systems may enable this.

## 1 INTRODUCTION

Across Europe and the USA, around two‐thirds of people with dementia live at home (Willink et al., 2020) and most prefer to remain in their own homes for as long as possible (Evans et al., 2019). Paid home‐care workers can support this (Dempsey et al., 2016). Home care is mostly provided by independent businesses in the UK (Sutcliffe et al., 2021), with workers known by a variety of terms (Cooper et al., 2017); for this study, we will use the term ‘home‐care workers’. The home‐care worker role is commonly perceived as undesirable (Schneider et al., 2019), and resourcing these workers is challenging (Hughes & Burch, 2020), with high staff turnover (Skills for Care, 2020). Despite ideals that home care is meaningful and responsive to individual needs (Dawson et al., 2015; Keogh et al., 2018), it has been scrutinised, in the UK and Ireland, for inconsistencies in the quality of care provision and the working conditions for home‐care workers (Genet et al., 2011). Gaps in care provision are often bridged by the individual's family (Brodaty & Donkin, 2009; Cherry et al., 2019).

Sixty percent of UK people receiving home care are living with dementia (Carter, 2015). Home‐care support for dementia that incorporates person‐centred approaches is considered the ‘Gold standard’ (Love & Femia, 2015) with the focus on the individuality of the person and their interactions and relationships with others (Hoel et al., 2021; Kitwood, 1997). Home‐care workers are well placed to promote person‐centred care, although they often feel unsupported to carry out their roles effectively (Cooper et al., 2017; Leverton et al., 2021a, 2021b).

Care plans are documents intended to communicate information and inform decision‐making (Burt et al., 2014); yet, their use is relatively unstudied. Titles of existing publications, for example, from general practice: ‘Are we stripping the care out of care plans?’ (Bacon et al., 2017) or from dementia healthcare settings, ‘Who's actually gonna read this?’ (Drummond & Simpson, 2017), convey a sense that in healthcare settings, their value is unclear. Care plans have been described as onerous documents: repetitive, time consuming, sometimes incomplete and inaccurate (Drummond & Simpson, 2017) or ‘only as good as their creator’ (Hsu et al., 2019). Some care recipients are unaware of their existence (Bacon et al., 2017).

Home‐care agencies have a duty to maintain good governance. In England, the Health and Social Care Act 2008 (Regulated Activities) Regulations 2014 require (and the Care Quality Commission regulates) that agencies ‘maintain securely an accurate, complete and contemporaneous record in respect of each service user, including a record of the care and treatment provided’. Care plans are central to how home‐care agencies deliver care, though their utility and impact have not been studied in this setting. Social services and home‐care agencies collaborate to agree care plans with clients or their family members where they lack capacity to do so. As people living with dementia face communication barriers to guide their own care, the issue of how intentions and realities of care align are particularly pertinent in this population. In the current study, we considered how the delivery of home care compares to the agreed care plan for clients living with dementia, by examining care plans and ethnographic observational field notes of home‐care visits to clients living with dementia.

## 2 MATERIALS AND METHODS

### 2.1 Study design

The study was approved by London (Camden and King's Cross) National Research Ethics Service (NRES) (reference: 17/LO/1713). Full details of procedures are reported elsewhere (Leverton et al., 2021a, 2021b). We undertook a secondary qualitative analysis of the ethnographic data derived from this earlier study, alongside an analysis of care plans for the clients observed, collected during the original study but not previously analysed.

### 2.2 Setting and sample

#### 2.2.1 Participant observations

We invited 11 home‐care agency managers to participate. They were purposively selected to encompass both local authority and privately owned agencies from urban and semi‐rural locations (two in London, two in South and two in North England). Of the managers approached, six agreed for their agency to participate in the study. The managers discussed the study with staff and identified home‐care workers interested in participating, who, in turn, approached their clients and/or their family carers. Informed consent was obtained from the home‐care workers, clients living with dementia and their family carers who agreed to participate in observations within these agencies. Where the client living with dementia lacked capacity to provide informed consent, a personal consultee was consulted, as per the Mental Capacity Act 2005 (England and Wales).

Three non‐clinical researchers (ML, AB and JBD) from psychology and sociology backgrounds observed home‐care workers’ interactions with clients living with dementia, between September 2018 and March 2019. They conducted up to two familiarisation visits for each client, where researchers collected contextual information only (i.e., number of home‐care worker visits per week, etc.) as well as formal observation visits where they wrote more detailed field notes, directed by a semi‐structured observation guide. The researchers recorded their reflections of their observations ‘in the moment’; thus, observation notes were made contemporaneously to or as soon as practicable after the visit.

The researchers also collected client care plans from the agency manager. Care plans were filed in the agency offices, as electronic documents that were available to home‐care workers. Agency procedures varied regarding whether a copy was also held at the clients’ home and whether home‐care workers could access it on site via an electronic system or only as a document on the agency computer. There was also variation in how frequently plans were updated and between home‐care workers in how frequently they consulted them for individual clients. Plans contained information about care needs, illness and personal details relevant to care provision and information about the tasks that home‐care worker(s) were to complete during visits. Confidential information was redacted prior to analysis. Home‐care agencies were assigned ID numbers between 1 and 6. We pseudo‐anonymised participants’ names: Letter ‘A’‐ for home‐care workers, e.g., ‘Alison’, ‘B’‐ for clients living with dementia, e.g., ‘Brenda’ and ‘C’‐ for family carers, e.g., ‘Cameron’.

### 2.3 Data analysis

We used a Reflexive Thematic Analysis approach (Braun & Clarke, 2006, 2019, 2021). Taking a constructivist perspective, we considered the dynamic between the home‐care workers and the person living with dementia. This epistemological perspective is well suited to ethnographic studies involving direct naturalistic observations (Williamson, 2006).

We compared the care plans of clients with their ethnographic field note observations to explore how the care observed aligned with the care that was instructed by the care plan. One researcher (PB) led the analysis; first, by becoming immersed in the data for each case study, comprising the client care plan, the familiarisation and observation visits for each home‐care worker‐client dyad or triad where a family carer was also observed. PB made notes about the care plan instructions, tasks and personal information about each client and considered how these compared with the field note observations. All co‐authors were allocated a proportion of case studies to read and independently explored developing themes.

We noted patterns of meaning, understanding and empirical knowledge within case studies and then across the data (Bowen, 2009). We generated initial ‘codes’, which were labelled and grouped into broader concepts. These were used to identify themes and sub‐themes (Braun & Clarke, 2006, 2021). Theme development was inductive and recursive, to ensure themes were meaningfully coherent (Braun & Clarke, 2006, 2021). ML, AB and JBD, who collected the primary observations, aided interpretations by reflecting on these experiences during data analysis, and as a team, we reflexively considered how our backgrounds, in mental health nursing (PB and KHD), psychiatry (CC) and social science (AB, ML and JBD), shaped our interpretations (Braun & Clarke, 2021; Cherry et al., 2019). We regularly discussed findings, interpretations and reflections to ensure that the analysis remained true to the research question (Connelly & Peltzer, 2016).

## 3 FINDINGS

### 3.1 Description of data

ML, AB and JBD observed 16 home‐care workers supporting17 people living with dementia, for whom we also collected agency care plans. Home‐care workers were all female, with a mean age of 48.9 years. We included 3 men and 14 women living with dementia, aged between 61 and 96 years. Home‐care visits ranged from 15 min to 2 h and included observations of home‐care workers undertaking personal care tasks, meal preparation, medication management, companionship and everyday housekeeping tasks (see Table 1 for client and visit characteristics). The researchers observed 100 h of home care, generating 104 individual field note transcripts. Most home‐care workers were observed working alone, but some worked in pairs to support clients. Family carers were also observed in some visits.

**TABLE 1:** Client and client care characteristics

Client name (age)	Home‐care worker (home‐care provider)	Private‐ or local authority agency‐funded care	Visits per day and scheduled visit duration (per visit)	Client needs (as stated within the care plan)	Client's living situation
1. Barbara (80)	Avery (4)	Local authority	3–4 visits per day of 30–45 min	A, B, C, D, E, F,G	Lives with son
2. Brenda (93)	Alison (2)	Local authority	4 visits per day of 30 min	A, B, D, F, G, L	Lives alone
3. Betty (no data)	Angela (1)	Private	3 visits per day (3 h visit in the morning, others length unknown)	B, F, G, H	Lives alone
4. Bridget (94)	Alison (2)	Local authority	3 visits per day of 30 min	A, B, F, G, K, H, L	Lives alone.
5. Benji (84)	Alison (2)	Local authority	5 visits per week of 30 min and 2 × 3 h ‘sitting service’	A, E, J,	Lives with wife
6. Bernice (89)	Alison (2)	Local authority	2 visits per day of 15 min	B, G, F, H, L	Lives alone
7. Beth (85)	Alison, Amy, Alice and April (2)	Local authority	3 visits per day of 15–30 min (5 days) and a 5 and 3 h longer visit each week	A, B, C, D, J	Lives with husband
8. Bea (89)	Aysha (6)	Local authority	2 visits per day of 30 min	A, B, D, F, G, H, L	Lives alone
9. Bara (98)	Alexa and Aida (5)	Private	4 visits per day of 1 h	A, B, C, D, E, F, G, H, I	Lives alone
10. Beatrice (96)	Anna and Audrey (2)	Local authority	24‐h care package	A, B, C, D, E, F, K, L	Lives alone
11. Belinda (82)	Anya (4)	Local authority	10 visits per week of 30 min	A, B, D, F, G	Lives alone
12. Benita (88)	Annie (5)	Private	2 visits per day of 1 h (5 days) and 5 h/1 h (other days)	F, H.	Lives alone
13. Beverly (77)	Ashley, Abby and Alina (3)	Private	2 visits per day of 1 h	A, D, K, L	Lives with husband
14 Bonnie (84)	Ashley (3)	Private	2 visits per day (1.5 and 3 h)	A, B, E, F, G, H, L	Lives alone
15 Boris (77)	Alexa (5)	Private	2 visits per week of 2 h	A, C, D, G, H, J,	Lives with wife
16 Brian (61)	April (2)	Local authority	2 visits per day of 45 min and 2 visits per week of 3 h	A, D, E, J	Lives with wife
17 Bryony (99)	Amanda (6)	Local authority	4 visits per day of 45 min	A, B, D, E, F, G, H, L	Lives alone

Note

A. Personal care. B. Nutrition/Hydration C. Mobility support. D. Continence needs E. Social support F. Medication. G. Meal preparation H. Support I. Shopping J. Sitting service/Respite K. Get client up and/ or put client to bed. L. Household chores, (hoovering, washing‐up, etc.).

Table 2 lists the dates of the last review of care plans, together with the date range of observations conducted. We noticed variations between each care plan in terms of the length of the plan (4–22 pages) and how often the care plan was reviewed and updated (8/17 had been written or updated in the 4 months prior to the first visit), whilst for cases 1, 3 and 16, it had not been updated for nearly 2 years. In Table 2, we include excerpts from care plans illustrating how practical details of care, and information to personalise care was referenced in the plans. This illustrates that some care plans (e.g., cases 2 and 5) include relatively scanty information, others very detailed and personalised information (e.g., care plan 9, 12).

**TABLE 2:** Client care plans (details and excerpts)

Client	Dates of observed visits	Date of first care plan and (date of review)	Number of pages in care plan	Practical details of care (examples)	Information to personalise care (examples)
1. Barbara	31.01.19; 08.02.19; 12.02.19 and 18.02.19	26.04.17 (no review date)	6	Few details: ‘Assist with personal care, assist with breakfast, meals and drink. Assist to go to toilet’	‘Check she is eating with a spoon and not with her hands’; ‘not able to prepare meals‐ requires assistance’
2. Brenda	22.01.19; 29.01.19; 06.02.19;12.02.19; and 26.02.19	03.06.14 (13.11.18)	16	Few details: ‘Personal care, food, meds and drinks’	No personalised or additional non‐task information in the care plan
3. Betty	08.08.18; 20.08.18; 31.08.18; 07.09.18;14.09.18 and 12.1.18	31.10.16 (10.01.18)	18	Few details: ‘Assist with preparing and serving meals. Assist to administer medication; accompany to go on a walk’	‘Likes to chat about family’ ‘Lost confidence using the phone.’ Used to attend the choir’
4. Bridget	29.01.19; 05.02.19; 12.02.19; 19.02.19 and 26.2.19	30.09.16 (05.11.18)	8	Few details: ‘Personal care, food, drinks and medication visits’	‘Leave snacks and a cold drink in easy reach on trolley’; ‘2 choices of clothes to choose from even if she declines a wash’. ‘May not want a wash in the morning, if she has refused, please attempt again in the afternoon’
5. Benji	29.01.19; 05.02.19; 12.02.19; 19.02.19 and 05.03.19	16.08.17 (07.11.18)	6	Few details: ‘Personal care, meds and sitting service’	‘Has prostate cancer so passes urine regularly’
6. Bernice	22.01.19; 05.02.19; 12.02.19; 19.02.19 and 05.03.19	28.04.17 (16.02.18)	6	Numerous details: ‘Medication visit (full assistance)’, ‘Requires support of another person to cook meals’, ‘Needs reminding to finish drinks’	‘There were recent reports of being outdoors, at the time the phone was broken and she was asking neighbours to call her son’, ‘Not being able to contact (Her son) would have been difficult and upsetting…’. ‘NOMADS (medication) kept above oven’
7. Beth	12.12.18; 19.12.18; 09.01.19; 16.01.19; 22.01.19; 29.01.19; 30.01.19; 05.02 19; 12.02.19; 19.02.19 and 26.02.19	07.08.18 (24.10.18)	4 (6)	Few details ‘Assist with personal care’, ‘Check pressure area’, ‘apply cream if needed’	‘Can be very outspoken and gets upset easily’ No strategies provided to manage this
8. Bea	28.02.19, 05.03.19 (two visits) and 08.03.19	20.08.18	7	Few details: ‘Require support throughout the day with personal care, meal preparation and administering medication’	Many examples provided including: ‘I am a calm person and do not have an abrupt nature’, ‘I enjoy being at home watching television’, ‘I am usually in bed on arrival of the carer in the morning’, ‘I tend to have a poached egg with some coffee’. (for breakfast)
9. Bara	04.12.18; 05. 12.18;11.12.18; 12.12.18; 28.01.19; 23.01.19 and 12.02.19	24.09.12 (20.06.18) and 25.10.18 (medication updated)	22	Numerous details included that marry‐up with personalised information (see adjacent column): ‘To support … to continue living in her own home’, ‘feel supported to continue with exercise, socialising and activities that she feels able to manage’	Numerous examples of individualised routine and preferences included in the care plan: ‘Goes to bed around 10 p.m., she puts the electric blanket on 20 min before she goes to bed, then turns her blanket off to unplug it before getting into bed’, ‘Very independent … quite forgetful’ Comprehensive information about personal care routine, preferences etc. is included in the care plan
10. Beatrice	28.08.18; 04.09.18; 11.09.18; 17.09.18; 18.09.18; 02.10.18; 10.10.18; 15.10.18 and 29.10.18	03.10.17 (25.09.18)	10	Numerous details: ‘Needs assistance with everything as is now bed‐ bound‐ personal care, feeding, medication’	‘Porridge for breakfast, fresh apple juice, cup of tea with one sugar’. Many suggestions for meals for supper/tea ‘May get agitated and scream, shout and howl’. No suggestions provided as to how to deal with this other than: ‘Carers are not to respond to her straight away. Please risk assess the situation’
11. Belinda	01.02.19; 06.02.19 (Two visits); 09.02.19; 18.02.19; (two visits)	23.02.18 (handwritten text overwriting the typed care plan)	4	Few details: ‘Ensuring eating, drinking, medication’. ‘Assist with meal preparation and drink’, ‘Prompt medication’	No personalised information included on the care plan
12. Benita	12.12.18; 18.01.19; 23.01.19; 28.01.19	06.09.16 (19.11.18)	22	Numerous details: ‘Wishes to remain living in her own home’. (Emphasis on independence on all identified care needs)	Detailed background and life story information included on the care plan as well as times and details of routine. Additionally: ‘Memory seems to have deteriorated and she feels she must write everything down’. ‘Has had quite a lot of dizzy spells…been to see the doctor and doesn't seem to be a specific reason for it’. ‘Writes lots of prompts down’
13. Beverley	13.08.18; 15.10.18; 19.10.18; 27.10.18; 22.10.18; 08.11.18; 15.11.18; 23.11.18 and 28.11.18	19.02.18 (14.02.18)	2	Few details: ‘I would need help with personal care in the morning and in the evening’; ‘I need a full body wash every morning’, ‘Change pad, cream on bottom’, ‘Clean nightie every day’	Personalised information not included. (Client lives with husband who is present during visits)
14. Bonnie	07.11.18; 15.11.18; 23.11.18; and 13.12.18	04.07.18 (updated care plan of 10 extra pages on 08.07.18)	2 plus 10 ‐for updated care plan	Numerous details: ‘Support with personal care (bathing and washing), ‘Support with meals, medication, anxiety about finance. Risk information included: ‘Risk of absconding’, ‘Support getting to day centre once a week’	Detailed information about life story. Very detailed separate information about anxiety and triggers for anxiety as well as strategies that the home‐care workers can try when the client becomes anxious or distressed.
15. Boris	20.11.18; 27.11.18 and 11.12.18	04.07.17 (16.07.19)	20	Numerous details: ‘Wishes to remain living at home’. ‘Wishes to stay active’	Detailed information about past history and client's life story as well as preferences and routines: ‘Try to have a short walk every day’, ‘Finds reading/watching TV difficult’, ‘Likes to listen to music’
16. Brian	28.08.18; 09.01.19; 16.01.19; 30.01.19	08.08.17 (02.08.18)	6	Few details: ‘Support with showering’, ‘Assist with dressing and shaving’, ‘Sitting service to give wife a break twice a week’	Details about preferences for meals provided: ‘Favourite food is fish pie’, ‘Was a keen vegetable grower’
17. Bryony	26.02.19; 28.02.19; 05.03.19; 08.03.19	15.1.18 (handwriting on the care plan states care plan review is overdue ‐should be annually)	6	Few details provided: Practical details provided, e.g. ‘I require carers to fill a hot water bottle for me before bed’	Some statements about choices, which had a generic quality: ‘I can make my own choices and decisions’ and ‘not know dislikes when asked, I can independently express my dislikes’

### 3.2 Qualitative findings

We identified one overarching theme, *Care Plans as a starting point but incomplete repository*, with two sub‐themes. The first sub‐theme, *Person‐centred care planning,* describes how the degree to which plans were tailored and relevant to the client appeared to influence the extent to which they mirrored the care that was delivered. The second sub‐theme, *Filling in the gaps* describes how home‐care workers responded to gaps in information or instructions within care plans when they were insufficiently detailed or out of date. To illustrate the findings, supporting observation notes are presented, followed by researcher reflections (where made) in italics. Where the excerpt is taken from the care plan, this is explicitly stated.

#### 3.2.1 Care plans as a starting point but incomplete repository

Care plans are conceptualised as the central repository of client information, but as described above, many were not regularly updated. The home‐care workers we observed were mostly aware of their clients’ care tasks as they were accustomed to performing them. We did not observe home‐care workers directly consulting a written care plan, perhaps for this reason. In one agency, electronic records allowed home‐care workers to interact with the care plan during visits.

…Ashley says she needs to make a record of this and tell the office. She goes on her phone to log it on the system. Bonnie sits in her chair eating her lunch. (Field note—Case 14)

In practice, this was perceived by the researcher to disrupt care delivery. The researcher's *in the moment* reflection of this described the electronic system as ‘distracting’ the home‐care worker and seemed to:

Disturb the flow of her delivering tasks as she logs information as she goes along, rather than at the end of the visit.

Whilst home‐care workers were rarely observed to directly refer to the care plan during the visits, they usually seemed aware of changes made to the client's care. This knowledge was sometimes passed on through written records or notes within the home. For case 7, one home‐care worker (Amy) writes in the care record kept in the client's home, although with a lack of accuracy: on one occasion, the written entry was pointed out by another home‐care worker (Alice) to be incorrect:

Alice corrected what Amy had written and told her to cross it out and write the correct thing—e.g., Beth didn’t give herself medication as Amy had written, it was in fact Cameron who does this. (Field note—Case 7)

Information was often passed on informally. It was evident from observations that home‐care workers held a valuable repository of information to inform care delivery, acquired from daily contact with clients, other home‐care workers or the clients’ families.

For example, in the excerpt below, the home‐care worker regularly obtained information about how to support her client from his wife, who was usually present at the start of each visit:

Caroline (wife) and her son went out shortly after I arrived. I asked April what was going on with Brian and she told me he had a bad chest infection and had been critically unwell in hospital over Christmas. Caroline told her Brian is sleeping all the time and not eating or drinking. (Field note—Case 16)

Brian's deteriorated health and subsequent care needs had not been updated in his care plan in the previous 2 years (Table 2).

Similarly, we observed further situations where informal sharing of information took place between family carers and individual home‐care workers, outside of the care plan. In observations of case 15, informal notes were used as communication between the home‐care worker and family carer, instead of using the care plan or a more formal communication record visible to other care workers or the home‐care agency:

Alexa said that she and Boris’ wife use written notes to each other to communicate’ (for example, on the familiarisation visit, Alexa left a note asking Boris’s wife to contact the District Nurse [DN] and confirm if Boris had had his flu vaccination…). (Field note—Case 15)

One pertinent observation showed that a new home‐care worker on her first visit with a client learned about the client's care needs verbally from another home‐care worker in a joint shift (case 7).

Amy tells Ava that they always put two pairs of socks on Beth’ (Does not refer to care plan or explain reason for this). (Field note—Case 7)

Informal information sharing, rather than consultation of the care plan, may result in decisions or updates becoming lost, leading to subsequent inconsistencies in care provision. In the following example (case 10), the home‐care worker did not agree with a care decision made by social services for the client to remain in bed (due to deteriorated mobility) and appeared to feel that her views and those of the client had been overlooked.

Anna feels that after 1 week in bed, someone with dementia will lose all ability to do anything for themselves and fears Beatrice will not even have the strength to hold herself up or walk after being in bed for 1 week. She says that the decision to keep Beatrice in bed has “completely taken everything away from her” and feels there is nothing they [the care workers] can do about it. (Field note—Case 10)

Despite the client having a large team of home‐care workers and several involved family carers, this critical information about the client's change in mobility and care needs was not updated in detail in the care plan, other than a short sentence saying:

Needs assistance with everything as is now bedbound (statement not signed or dated). (Care plan—Case 10)

Other needs within the same care plan were not amended to reflect these changes:

At best walks with a frame, at worst, transfers with stand. (Care plan—Case 10)

##### Sub‐theme A—‘Person‐centred care planning’

In this sub‐theme, we explored whether individualised information about the client was contained within the care plan and whether this impacted the care that was delivered. In several cases, more up to date and personalised care plans included information specific to the individual client, such as their likes, dislikes and functional abilities, and suggestions for how to provide care that promoted choice and independence. In these instances, this person‐centred advice was often reflected in practice. For example, the care plan for Bara (case 9) had been updated 2 months prior to the observation visits; the home‐care workers were guided to involve Bara in choosing what she wanted to eat:

‘Bara needs all her meals prepared for her’ … ‘Bara can make decisions with support’. (Care plan—Case 9)

This was reflected in observations of the home‐care worker, Alexa, supporting Bara to make decisions around meal planning by offering her choice:

Alexa takes the plate and asks Bara if she fancies a dessert. She offers Bara three choices from which Bara chooses trifle. (Field note—Case 9)

When information about the client's personality or life story was included in the care plan, the home‐care worker was seen to use this knowledge (which may have been obtained from the care plan or other sources) to encourage conversation and social interaction with the client:

Boris spends a lot of time listening to TV. He likes sport and gardening (Care plan—Case 15)

Alexa brings into conversation that Boris used to be a keen vegetable grower … he tells us about his greenhouse and how it was heated … Boris joins in about what his wife likes to do to the lawn to make more room for cuttings. (Field note—Case 15)

In the following example, the inclusion of tailored, personalised suggestions in the care plan, such as strategies to manage the client's memory loss, may have been helpful to home‐care workers:

Benita’s memory appears to have deteriorated, and she feels she must write everything down … . Likes to rely on the caregivers to remind her of daily tasks, this includes writing reminders in her purple diary next to her armchair. (Care plan—Case 12)

The home‐care worker, Annie, was observed also using the purple diary to communicate information to other home‐care workers who support Benita:

Annie updates the diary in the kitchen (that all the care team and Benita read and input to), to record the neighbours visit and she tells me that events that are important like people visiting, or telephoning go in the diary to help Benita remember they have happened and to keep track of when she last saw or spoke to someone. (Field note—Case 12)

In another example, tailored information about how to manage the client, (Bonnie)’s, anxiety provided a clear plan for the home‐care workers to follow. The client's care plan had recently been updated and highlighted the specific triggers/factors which caused her to become anxious, distressed or confused, detailing specific instructions for the home‐care workers to follow when Bonnie becomes anxious:

‘Ask (Bonnie) to look at her black and red purses and prompt (Bonnie) to look at her savings book in her bag. Also, please remind her during this stage that she owns her home and has 3 bank accounts and in one of them she has a substantial amount (which daughter is keeping safe). 1 savings account is in the bag, 2 accounts her pension goes in, and this account pays her bills (water, electricity, gas and phone). She does not pay for TV license and council tax.’ … ‘Always keep reassuring (Bonnie), tranquil body language and effective communication. Attempt to go along with what she says to avoid further anxiety.’ (Care plan—Case 14)

This was observed in practice during visits when Bonnie became quiet and seemingly worried about what the home‐care worker, Ashley, was doing. The home‐care worker was able to provide reassurance:

Ashley tells Bonnie that there is nothing to worry about in terms of the finances, she is just trying to send the reading to the company. (Field note—Case 14)

By contrast, there were multiple examples of times where more tailored information about the client's social network and relationships or advice around how to manage challenges was not provided in care plans but might have been helpful to home‐care workers in particular situations. This seemed to be more evident with care plans that had not been updated in the past year. For example, in the care plan for case 11, there was no mention of the client's pet cat, to inform the home‐care worker, Anya, of the client's relationship with her cat, or what to do during visits:

The cat comes in and goes near Belinda. Anya shouts at the cat to get away from Belinda and her dinner and says I don’t like cats. Anya gently kicks the cat away. (Case 11)

A number of care plans included generic statements about dementia, rather than more specific, tailored advice as in the examples above. For example:

People with dementia can become apathetic and uninterested in their usual activities … they may also lose intent in socialising. (Care plan—Case 8)

In practice, the home‐care worker, Aysha, and client, Bea, (case 8), were regularly observed engaging in social conversation, and as in the example below, the client initiated and engaged the researcher in conversation. Bea's ability or desire to socialise was not described in the care plan as the information provided was not tailored to her as an individual:

The conversation is light‐hearted and covers a variety of topics and Bea includes me [the researcher]in their conversation about holidays that she is going on, reminiscing about the work she used to do, and how she likes singing and dancing. (Field note—Case 8)

In the following observation between a home‐care worker, Audrey, and her client, Beatrice, Beatrice's mobility had deteriorated (as described in an earlier section above), and her needs had changed in the preceding weeks, resulting in her care being completely provided in bed. The researchers observed that a mattress was placed on the floor to cushion any potential falls:

The room is also cramped because of the fall mattress that is next to Beatrice’s bed and takes up the majority of the floor space. Audrey visibly struggles to move around this and avoids standing on it. (Field note—Case 10)

However, the care plan had not been updated to reflect the client's change in situation or how this might impact on care provision.

##### Sub‐theme B—‘Filling in the gaps’

In this second sub‐theme, we explored how home‐care workers used their intuition, skills and knowledge of the client to compensate for missing care plan information. With case 13, the care plan states that the client's husband (Cameron) assumes responsibility for most of his wife's care needs, except for personal care tasks which the home‐care workers undertake in the morning and evening. However, there are no instructions for the home‐care worker to follow when care needs beyond those stated within the care plan, arose:

My husband manages everything related to my care. He looks after our house, prepares food and drink, does the shopping, administers my medication and deals with my laundry. (Care plan—Case 13)

In one example, observation with this case, Abbey notices Beverley is thirsty but appeared to have a trial‐and‐error approach to getting her to drink:

Abbey seems to have found a way to encourage better intake of drink from Beverley … Abbey asks Beverly to take a sip. Beverly does not respond initially when Abbey holds the bottle up to her mouth. She lowers it and Beverly opens her mouth. Abbey puts the bottle to Beverly’s mouth and Beverly takes a number of sips. She drinks a lot more than when [a different home‐care worker] has given her the juice in previous visits—usually only two sips and then she puts the bottle back.

In this example, Abbey responded intuitively and successfully gave the client a drink, but as the care plan was not updated to include any strategies, this information was unavailable to other home‐care workers.

In another example of a care plan that was relatively vague and less specific to the individual client's needs, Beth's care plan instructed home‐care workers to:

Assist with personal care, change pad, check pressure area, reposition, apply cream if needed. (Care plan—Case 7)

However, there was no guidance about where the pressure areas are or where to apply cream. Beth's care was always provided by two home‐care workers, although this was not stated in the care plan. Without clear instruction of the roles that both home‐care workers should take during visits, or how they should work together, the home‐care workers routine appeared unorganised, without clarity of who the ‘lead’ care worker was:

Alison commented that it was dark, and April walked around the bed to turn the light on. April stood on the left side of Beth’s bed and Alison said to April that she should be on the other side of the bed [the care worker on the right side of Beth predominantly does the pad change and wash while the care worker on Beth’s left supports Beth while she leans to one side]. April said that she was staying where she was, and that Alison should be on the left side because she was the more senior carer. (Field note—Case 7)

Beth did not have a consistent team of home‐care workers; therefore, the lack of direct instruction within the care plan as to ‘who is doing what and how’ may have hampered care delivery. On several occasions, the researcher observed Beth becoming distressed during personal care tasks:

Both April and Alison spoke at the same time giving many instructions such us "move this leg here, bend this knee and move that leg” and Beth looked confused and irritated. Beth said she just wanted to be left alone. (Field note—Case 7)

Whilst the care plan records that Beth is ‘very outspoken and gets upset easily’, it does not provide strategies on what works to reduce her distress.

In contrast, as shown in Table 2, other care plans gave a greater level of detail. The following care plan included clear, comprehensive instructions around the client's personal care needs, which seemed sufficient to ensure consistency in care delivery in practice:

Bring a dining chair into the bathroom ready for Bara to sit on for drying after her shower. Walk in with Bara to the shower leaving her trolley at the bathroom door. Help Bara to take her nightwear off. Place non‐slip mat in the shower floor and run the shower to Bara’s preferred temperature. Guide her hand until she has hold of the grab rail in the shower and has lifted her feet over the step‐mainly for confidence. Bara will wash the front of her body, caregivers to assist with washing back, legs and feet. (Care plan—Case 9)

## 4 DISCUSSION

To our knowledge, this is the first qualitative study to explore how care that is provided by home‐care workers for clients living with dementia compares to written care plans. Our overarching theme—*Care plans as a starting point but incomplete repository* described how care plans were in practice static documents and often not up to date. There was, thus, a network of information exchange, between home‐care workers and with clients and families that they did not formally record. Four of the care plans had not been updated for 2 years, so were very unlikely to remain relevant, particularly with individuals living with dementia for whom care needs can progress faster than people without dementia. Most of the examples of specific and detailed care plans that could enable person‐centred care had been updated in the past 4 months prior to the observation visits.

We identified two sub‐themes: (a) *Person‐centred care planning*, which explored the degree to which the care plan reflected the individuality of the person living with dementia, and (b) *Filling in the gaps*, which explored how, where there was a lack of detail in plans, home‐care workers ‘filled in the gaps’: often creatively, but with a lack of assured consistency between home‐care workers or ability to learn from trial‐and‐error, as these creative strategies were generally unrecorded in care plans.

Previous research has suggested that the inclusion of meaningful biographical information in the client's care plan is crucial to develop an understanding of how the person perceives what is going on around them (Hughes & Burch, 2020) and potentiates a person‐centred approach that can be enhanced by collateral information from family (Molony et al., 2018). Comprehensive and individualised care plans have been promoted as more likely to uphold personhood in dementia (Hennelly et al., 2021), help to minimise risks from unmet needs (Molony et al., 2018), are more likely to enhance autonomy (Lambert, 2019), and sense of self of individuals living with dementia (Førsund et al., 2018). Our findings seem to accord with this. We report examples where care plans were up to date and tailored to the current care situation, and the care plan appeared to concord with the care delivered in practice. In other instances, the converse was true, highlighting the need for care plans to be updated regularly, particularly for people living with dementia where symptoms and deterioration can progress quickly.

The need to ‘fill in the gaps’ could lead to home‐care workers experiencing increased stress and a greater sense of accountability for their actions; as described previously, home‐care workers experienced considerable stress, related to feelings of onerous responsibility for clients (Leverton et al., 2021a, 2021b).

Our findings suggest that home‐care providers could usefully re‐evaluate how they use care plans and set standards for their regular review to promote consistency of care. This may involve introducing digital solutions such as electronic care records, currently used within many residential care homes (Shiells et al., 2019). Whilst digital care approaches such as health portals document and exchange client information between stakeholders as a dynamic process (Guisado‐Fernandez et al., 2019), technological options have not been fully integrated into home‐care practice. Where we observed the use of electronic records in one agency, it appeared to distract the home‐care worker and disturb the flow of care provision. Digitalisation of home‐care records and care plans is, thus, unlikely to be straightforward, and further research to explore how it can be implemented and used by home‐care workers may be important to enhance care quality, rather than create an administrative burden that distracts from care tasks.

Our findings suggest that inclusion of information about how care is best provided to individual clients as well as what care is provided, may enable good quality care by sharing good practice. We acknowledge, however, that the development of care plans needs to balance including helpful detail with being sufficiently succinct to be accessible. Professional home care can be stressful even there are appropriate and wisely used care plans.

### 4.1 Strengths and limitations

Whilst this was the largest observational study to date in home care, the sample may not be representative of all home‐care clients or providers. Whilst we speculate that more detailed, person‐centred care plans enabled more person‐centred care (and these factors seemed to be linked), it is also possible the home‐care agencies which have greater resources, or better management, review care plans more regularly and are more facilitating of person‐centred care in other ways, through staff working conditions and training, for example. The potential impact of the observers of the naturalistic environments they sought to observe must also be acknowledged.

## 5 CONCLUSIONS

In the context of dementia, the associated complexities of the condition, where the client's needs can rapidly change, can render care plans redundant unless they are designed to be flexible, regularly updated and responsive to the condition. This study concludes that where care plans are vague or lacking in person‐centred client information, the need for home‐care workers to ‘fill in the gaps’ is increased, risking inconsistent care provision and home‐care worker stress. A comprehensive care plan that is readily available to the home‐care worker can enhance and support a person‐centred approach, contribute to better care for people living with dementia and enhance working conditions for the home‐care workers.

## CONFLICT OF INTEREST

The Author(s) declare(s) that there is no conflict of interest.

## Data Availability

Suitably anonymised data are available from the corresponding author on receipt of an appropriate request.
